# Effect of tailored, intensive prehabilitation for risky lifestyles before ventral hernia repair on postoperative outcomes, health, and costs – study protocol for a randomised controlled trial (STRONG-Hernia)

**DOI:** 10.1371/journal.pone.0324002

**Published:** 2025-05-28

**Authors:** Sofie Anne-Marie Skovbo Jensen, Susanne Vahr Lauridsen, Siv Fonnes, Jacob Rosenberg, Hanne Tønnesen

**Affiliations:** 1 WHO-CC/Clinical Health Promotion Centre, The Parker Institute, Bispebjerg and Frederiksberg Hospital, Frederiksberg, Denmark; 2 Department of Surgery, Centre for Perioperative Optimization, University of Copenhagen, Herlev Hospital, Herlev, Denmark; 3 Department of Urology, University of Copenhagen, Herlev Hospital, Herlev, Denmark; 4 Department of Clinical Medicine, University of Copenhagen, Copenhagen, Denmark; 5 Department of Health Sciences, Faculty of Medicine, Lund University, Lund, Sweden; Manipal College of Health Professions, INDIA

## Abstract

**Background:**

A substantial untapped potential for risk reduction may be fulfilled by applying intensive lifestyle interventions targeting the co-existing risky lifestyle factors Smoking, Nutrition (both malnutrition and obesity), risky Alcohol intake, and Physical inactivity (SNAP) before surgery. This trial will compare the effect of combined and individually tailored prehabilitation with standard care on postoperative outcomes, health, and cost-effectiveness in short and long term in participants undergoing ventral hernia repair. An interview study will be nested within the randomised trial.

**Methods:**

The study is a multicenter, parallel-group, superiority randomised clinical trial. A total of 400 adult participants undergoing ventral hernia repair with ≥1 SNAP factor will be allocated to the individually tailored STRONG programme or standard care. The STRONG programme is initiated at least four weeks prior to surgery and consists of six sessions. It is delivered as one session a week, approximately, and includes patient education, motivational, and pharmaceutical supports. The primary outcome is postoperative complications requiring treatment within 30 days. Secondary outcomes address other surgical outcomes, changes in lifestyle, health, and cost-effectiveness. Follow-up takes place after 6 weeks (the end of intervention), at surgery, and 30 days, 90 days, and 6 months after surgery, respectively. Long-term data on health and costs will be obtained from nationwide registries after two years. Eligible trial participants will be invited to a semi-structured interview study at baseline. Their reflections on the STRONG programme and the choice of participating in the trial or not will be explored.

**Discussion:**

Many patients have multiple SNAP factors adding to the risk of complications related to surgery. As these are modifiable, prehabilitation may be an area with great potential for risk reduction. Nevertheless, no well-acknowledged and evidence-based strategies exist in the preoperative period. The STRONG programme is tailored specifically to the individual patient’s preidentified needs including up to all five common risky SNAP factors and may tap into the large unused potential for risk reduction. Overall, the study will add important new knowledge on the effect of individually tailored prehabilitation on complications and other important outcomes in elective surgery, and also clarify if this intervention will have long-lasting implications.

**Trial registration:**

www.clinicaltrials.gov (NCT06611462).

## Introduction

### A general problem to be solved

Surgery may be associated with postoperative complications and the common risky lifestyles Smoking, Nutrition (both obesity and malnutrition), risky Alcohol intake, and Physical inactivity (SNAP) are known to contribute to the risk of these in varying degrees. Daily smoking [1] and alcohol intake >2 drinks/day [2] each increases the risk of postoperative complications by around 50% whereas severe malnutrition [3], obesity [4,5] and physical inactivity [6,7] add to the risk in a varying degree.

The surgical pathway has been optimised with the Enhanced Recovery After Surgery (ERAS) [8,9] and improvement of surgical and anaesthesiologic techniques over the years. However, there is still a large untapped potential for risk reduction in the preoperative period where the modifiable SNAP factors can be targeted with preoperative interventions commonly known as prehabilitation. For smoking and alcohol, intensive programmes aiming at complete cessation four to eight weeks before surgery have been shown to reduce postoperative complications by up to 50% [10,11]. Likewise, preoperative nutritional interventions for patients with severe malnutrition have also shown a reduction of postoperative complications by around 50% [12–14]. Weight loss interventions in obese patients have only been sparsely investigated in other areas than bariatric surgery [15]. Preoperative physical training programmes have shown improvement of physical capacity. While most studies have not demonstrated a significant impact on postoperative complications [16,17], one recent study suggests a possible benefit [18]. The majority of the prehabilitation evidence comes from studies only investigating one SNAP factor at a time [19] despite that up to around half of hospital patients have co-existing SNAP factors adding to their surgical risk [20,21].

The STRONG programme investigated in this trial will be tailored to and offered based on the individual participant's preidentified need for lifestyle-related risk reduction and will include up to all five risky SNAP factors if present. To the best of our knowledge, it will be the first programme to combine all five risky SNAP factors. Furthermore, our recent systematic review on multimodal prehabilitation [22] has shown that close to none of the published studies have had a demand for a pre-identified lifestyle-related need of risk reduction when offering prehabilitation. It also showed that most prehabilitation research has been conducted in cancer and other major surgery. Therefore, this trial will be a pioneer study regarding prehabilitation offered based on a pre-identified need as well as prehabilitation before minor surgery.

### Ventral hernia repair and SNAP factors

Ventral hernia repair is one of the most common surgical procedures [23] and its benign nature allows time for preoperative optimisation to reduce postoperative complications [24]. Surgical site infections (SSIs) more than double the risk of ventral hernia recurrence [25], and since obesity and smoking are believed to be risk factors for SSIs, prehabilitation at least addressing these factors should be an obvious focus for research [26]. Nevertheless, limited research exists on SNAP prehabilitation. A recent systematic review on lifestyle prehabilitation before ventral hernia repair shows that the amount of research conducted is sparse [27]. Two randomised controlled trials (RCTs) on preoperative smoking cessation were identified [28,29]. Only the RCT investigating an intensive intervention showed a significant result with halving of complications (mainly wound complications) [28]. One study on combined obesity and physical activity intervention was identified. Short-term results showed a lower seroma rate at 30 days in the prehabilitation group and they were also more likely to be complication- and hernia-free [30] but there was no difference between the two groups at long-term [31]. No studies were identified on nutrition/malnutrition, but five studies were identified on a low-calorie diet’s effect on weight loss in obese/morbidly obese patients [32–36]. However, none were RCTs and only one commented on postoperative complications. Finally, alcohol intervention was not evaluated in the review.

Overall, there is a call for prehabilitation including more SNAP strategies, and the European Hernia Society strongly recommends conducting high-quality studies of prehabilitation before ventral hernia repair [27].

### The frameworks

Two frameworks are integrated in this study:

- Prehabilitation entailing preoperative recovery of dysfunctional organ systems prior to surgery [37,38]
- Lifestyle interventions being introduced by the operational model (based on principles of motivational interviewing, balanced decision-making, and the stages of change model) [39] and delivered as the Gold Standard Programme (GSP) for smoking cessation [40] and adapted versions for the other risky SNAP factors

### Objectives

The main objective is to investigate the effect of the STRONG programme – a tailored, intensive SNAP prehabilitation programme – prior to ventral hernia repair for participants with at least one SNAP factor on postoperative complications requiring treatment within 30 days compared with treatment as usual. Secondly, we aim to investigate the effect of the programme on other surgical outcomes, changes in lifestyle, health and cost-effectiveness on short and longer term, as well as participant reflections and preferences.

The main hypothesis is that postoperative complications requiring treatment within one month can be halved by the STRONG programme compared with treatment as usual.

## Materials and methods

### Trial design and setting

**Fig 1:**
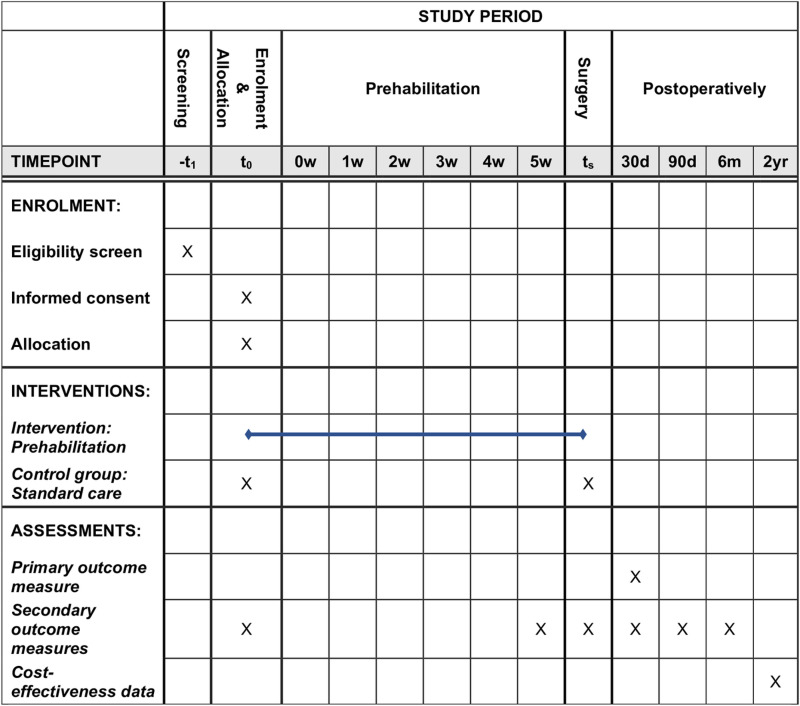
Schedule of enrolment, interventions, and assessments. d : days, m: months, -t_1_: time point for screening before enrolment, t_0_: time of enrolment, t_s_: time of surgery, w = week, yr = years.

The trial is a multicentre, parallel-group, superiority RCT with an allocation ratio of 1:1, see Fig 1. Furthermore, the project uses other nested relevant study designs to test the hypotheses and answer the research questions including qualitative methods [41], costs, and secondary analyses.

Participants will be recruited from three surgical departments in Denmark: Herlev Hospital, Zealand’s University Hospital in Køge, and Zealand’s Regional Hospital in Holbæk. More departments may be included if necessary.

The intervention takes place at the hospital from which the participants are included. It is carried out by local project nurses and staff from the participating centres certified by a training course carried out by the STRONG-team. The STRONG-team is anchored at the WHO-CC, Clinical Health Promotion Centre at the Parker Institute, Bispebjerg-Frederiksberg Hospital, part of Copenhagen University Hospital in Denmark.

The trial is registered at www.clinicaltrials.gov (NCT06611462). Reporting of this study protocol followed the SPIRIT (Standard Protocol Items: Recommendations for Interventional Trials) guideline [42].

### Eligibility criteria

#### Inclusion criteria.

Participants ≥ 18 years old scheduled for elective ventral hernia repair with a defect < 8 cm and having at least one of the five risky SNAP factors:

- Smoking: daily smoking (defined as ≥1 cigarette or the equivalent of 1 gram of tobacco daily)
- Obesity: BMI ≥ 30 kg/m^2^
- Malnutrition: Nutritional Risk Score [43] (NRS) ≥ 3
- Risky alcohol intake: Above 14 units/week during the last month (1 unit is defined as being equivalent to 12 grams of alcohol) [44]
- Physical inactivity: <30 minutes of physical activity per day or < 3.5 hours per week [45]

Upon inclusion, expected time before surgery must allow enough time for at least 4 weeks of prehabilitation.

### Exclusion criteria

#### Exclusion criteria include.

Other ventral hernias (para-stomal hernias and giant ventral hernias with defect ≥ 8 cm); pregnancy and/or breastfeeding; allergy or other contradictions to pharmaceutical or nutritional support or exercise in the STRONG programme; not able to give informed consent to the research project (e.g., age < 18 years, severe mental illness, compromised consciousness, language challenges); previous alcohol delirium or seizures; or withdrawal of consent.

### Interventions

#### Intervention group.

**Fig 2:**
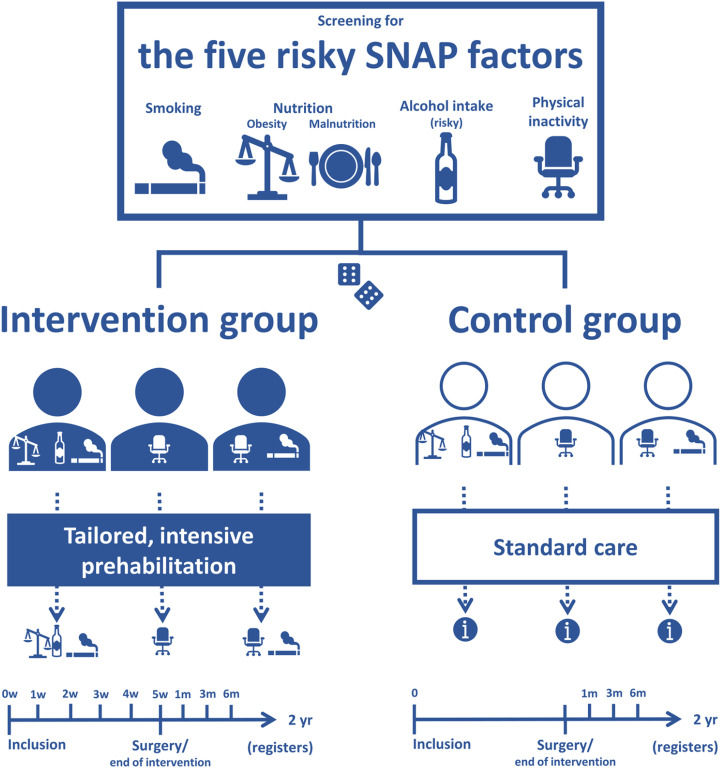
An overview of the STRONG-Hernia trial conduct. The figure illustrates the screening for the five risky SNAP factors (Smoking, Nutrition (obesity and malnutrition), risky Alcohol intake, Physical inactivity), the randomisation into the intervention and the control group, and how the intervention group (blue persons) receives a tailored, intensive prehabilitation intervention on exactly the risky SNAP factor that they present with whereas the control group (white persons) receives standard care. At the bottom of the figure is a timeline for scheduled visits and follow-ups in each group, respectively. i = information and demands for each hospital is: Herlev Hospital, generalized preoperative optimization including smoking and BMI (no cut-off); Zealand University Hospital, information on weight loss and smoking cessation with telephonic follow-up by nurses (aim of BMI < 35 and smoking cessation is mandatory); Zealand Regional Hospital, aim of BMI < 35 and no demand or help regarding smoking cessation. m: months, w: week, yr: years.

Participants in the intervention group receive the intensive prehabilitation STRONG programme prior to surgery which is tailored to meet the individual participant’s need for lifestyle improvement (Fig 2) and thereby potential risk reduction at surgery. The programme includes a minimum of six sessions distributed over approximately six weeks (i.e., about weekly) including patient education, motivational support, and pharmaceutical support, such as nicotine replacement when indicated, and a hotline where the participants can contact the project staff. The education is directly related to their surgical treatment and includes 1) an introduction to the STRONG programme, level of motivation, ambivalence, pros and cons; 2) symptoms of addiction and/or withdrawal symptoms including their experiences and expectations; 3) relapse description and management; 4) benefits of lifestyle change on short and long term; 5) continued change of lifestyles including handling the risk of relapse; 6) and continued education based on the current conditions at the late stages in the programme.

**Fig 3:**
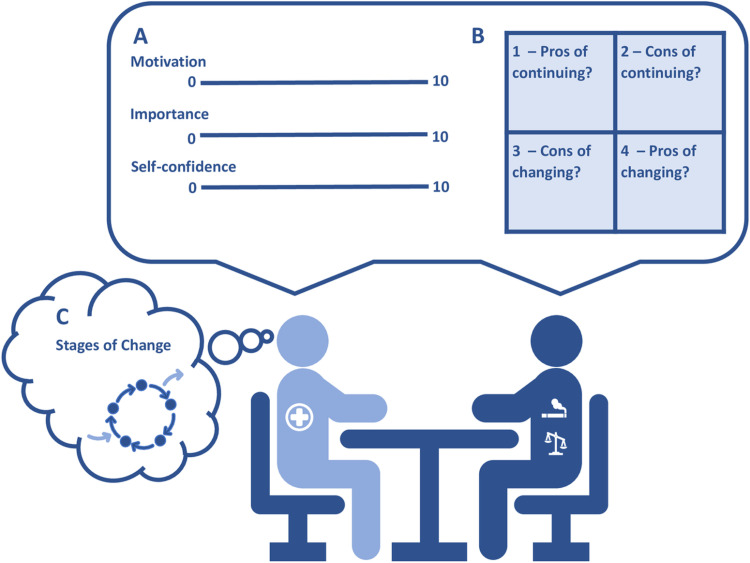
Behavioural change theories. Health care professional introduces behavioural change to a participant with risky lifestyles using A) the line tool through motivational interviewing and B) the box tool to make the participant reflect on changing lifestyle. Meanwhile, the health professional uses C) the circles in the stages of change model to evaluate where in the process of changing lifestyle the participant is.

It is introduced with the surgical recommendations in “Engage in the process of change” with the operational model [39]. It combines three behavioural change theories (Fig 3): motivational interviewing (the line tool) [46], decisional balance (the box tool) [47], and the transtheoretical model of change (the circles) [48]. The line tool: Participants will be presented with three lines with numbers each ranging from 0–10. Each is introduced with a statement setting the scene and then a related question making the participants reflect and rank their motivation (line 1), priority (line 2), and self-believe (3) in changing their lifestyle before the surgery. The box tool: Consists of a box divided into four sections asking the participants to fill out pros and cons for changing their current lifestyle or continuing it, respectively. The circles: A model describing how changing addictive behaviours occurs in stages and how recycling through the stages of change several times is a natural part of the progress towards quitting an addictive behaviour. The combination of the three theories facilitates a successful lifestyle change by facilitating the dialogue about lifestyle change, making the participant reflect on their ambivalence with pros and cons of changing/not changing their current risky lifestyle, inducing a feeling of empowerment to reach a decision, and achieving a better understanding overall of the process of change.

The smoking cessation intervention follows the Gold Standard Programme GSP [40] whose principles and structure have been used for creating the nutritional intervention, the alcohol cessation intervention used in previous RCTs [49,50], and the physical activity intervention. The focus of the STRONG programme generally lies on healthy days, i.e., days without smoking or alcohol intake, eating according to the nutritional plan and being physically active according to the plan. The number of SNAP factors involved is according to the individual participant’s profile of risky lifestyles. For smoking, personalised nicotine replacement therapy based on the internationally acknowledged Fagerström test [51] for nicotine dependency and participant preferences is offered. The nutrition programme includes an individual assessment of the nutritional needs of the participant resulting in a nutritional plan targeting malnutrition and/or obesity. Participants with a risky alcohol intake are offered pharmaceutical support including thiamine (300 mg daily) and combined B vitamins, alcohol withdrawal prophylaxis and treatment (chlordiazepoxide 10 mg) and a low dose of disulfiram (200 mg × 2 weekly, administered only if the participant has a negative alcohol breath test). The pharmaceutical support for smoking and alcohol cessation interventions follows the national recommendations. All pharmaceutical support is an offer and not a requirement for participating in the intervention. It will be offered for free during the intervention period. The physical activity intervention consists of five minutes of inspiratory muscle training (IMT) and 25 minutes of other physical activities, adding up to 30 minutes per day. The IMT needs to be trained every day for the participants to be compliant, but the other physical activities can differ in time between days as long as the total amount of IMT and other physical activities add up to a total of minimum 3.5 hours per week. Furthermore, all participants in the intervention group will receive oral immunonutrition supplements in the five days up to surgery which both provide nutrition and boost the immune system [52]. The entire programme is adapted to the individual participant including guidance on supportive medicine. The STRONG prehabilitation will be considered completed if the participant has attended at least 75% of the six meetings.

At each meeting, we look at the current status and follow up using logbooks with self-reported information by the participants on the intervention since the last meeting as well as provide the motivational support and the patient education session. Participants are asked if they have experienced harms related to the pharmacological support. All potentially unknown harms will be reported and if serious they may lead to the trial being terminated early. The control group receives the project information as well as standard care of the involved departments, see Fig 2 (e.g., smokers may be offered a Very Brief Advice and referral to a municipal clinic). They also all have telephone access to the research nurse during the project. All receive routine procedures on general patient information, thromboembolic prophylaxis and antibiotics, anaesthesia, surgical intervention, and postoperative care as used at the centres.

#### Control group.

Comparators will be equal to the participants in the intervention group, i.e., having one to five risky SNAP lifestyles and undergoing elective ventral hernia repair, but receiving standard treatment in the preoperative period according to local routines instead of the intensive STRONG intervention (Fig 2). The control group will receive brief advice regarding the SNAP factors as part of the current standard protocol for surgical patients in the respective departments participating in the study.

The control group will receive the same project information about the study as the intervention group. Furthermore, they will receive the same routine procedures on general patient information, thromboembolic prophylaxis, antibiotics, anaesthesia, surgical intervention, and postoperative care used at the centres as the intervention group. Standard treatment regarding the SNAP factors for the involved hospitals is very brief information on weight loss and/or smoking cessation (Fig 2).

All participants in both groups are allowed to seek and use any support available outside the project, e.g., free municipal guidance on lifestyle change.

#### Criteria for discontinuing or modifying allocated interventions.

Participant withdrawal from the study is an option at any point and for any reason without impacting any future investigations and/or treatments at the site. The investigator may discontinue any participant’s participation for any reason, e.g., an adverse event, safety concerns, or failure to comply with the protocol. The study may be terminated by the principal investigator at any time. Reasons include but are not restricted to unsatisfactory fulfilment of the design, enrolment of participants, keeping the time schedule, or administrative agreements.

#### Strategies to improve adherence to interventions.

Adherence to the intervention is measured as meeting adherence registered during the intervention and follow-ups. The motivation work conducted at each meeting and the 24/7 hotline should help keep up meeting adherence and improve the number of healthy days the participants have between the meetings. Participants will fill out logbooks at home regarding their risky lifestyles, and the lifestyle changes will then be monitored by interviews at the meetings and validated by objective measures as well as blood and urine markers. The general interest among participants regarding their personal results of measurements and monitoring may further improve adherence to intervention.

### Outcomes

**Fig 4:**
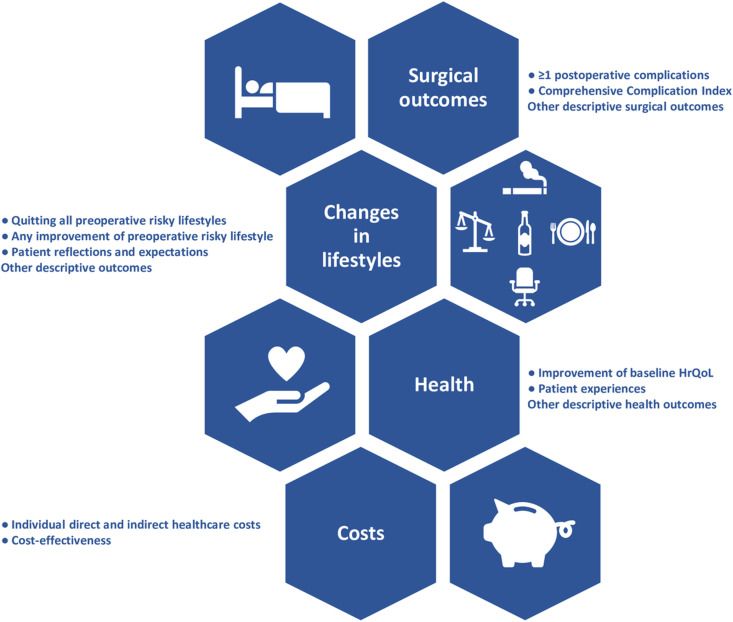
Overview of the four clusters of outcomes in STRONG-Hernia. HrQoL: health-related quality of life.

No core outcome set exists for trials on prehabilitation before elective surgery or ventral hernia trials, but a new scoping review gives an overview of the different outcomes reported in randomised trials of surgical prehabilitation [53]. Many of the outcomes reported in this trial will be consistent with some of the most commonly reported outcomes in other prehabilitation RCTs across different kinds of surgery. Four clusters of outcomes will be evaluated: surgical outcomes, changes in lifestyles, health, and cost (Fig 4). All outcomes will be evaluated within the intervention and control group separately, if not stated otherwise.

#### Surgical outcomes.

The primary outcome is the number and proportion of participants with ≥1 postoperative complication requiring treatment within 30 days after surgery. This will also be evaluated at three and six months postoperatively as secondary outcomes. The complications will also be categorised according to the Clavien-Dindo classification [54].

Secondary outcomes also include the Comprehensive Complication Index (CCI) [55] at 30 days, three, and six months postoperatively. The CCI is calculated from the Clavien-Dindo classification [54] of the postoperative complications. Hernia-specific complications such as hernia recurrence will also be reported with number and proportion at 30 days, 90 days, and six months. Participants will undergo a physical examination at all postoperative follow-ups in addition to questions about complications.

Other pre-specified outcomes include postoperative length of stay (LoS) defined as the total time spend in-hospital from the end of surgery and within the first 30 postoperative days, number and proportion of participants with readmission within 90 days, and the time back to work/usual activities asked at 30 days, three months and six months follow-up. Both LoS and time back to work/usual activities will be presented as median and/or mean. The number and proportion of participants with any visits to primary care at 30 days postoperatively will be calculated based on self-reported data from the 30-day follow-up.

#### Changes in lifestyles.

The outcomes include the number and proportion of participants who eliminated all pre-operative risky lifestyles and those who showed any improvement compared to baseline. Both outcomes will be evaluated at the end of intervention, surgery, 30 days, three months, and six months postoperatively. For the separate SNAP factors, quitting a risky lifestyle is defined by:

- Successful smoking cessation defined as no use of any tobacco products at any time, validated by carbon monoxide (CO) in the breath test and urine-cotinine (u-cotinine)
- Malnutrition: not at risk of malnutrition defined by NRS < 3 [43]
- Obesity: BMI < 30 or 5–10% loss of body mass compared to baseline [56] without developing malnutrition (due to loss of fat-free mass) [3] at end of intervention/surgery and below 1% gain of body mass (as fat mass) at postoperative follow-ups
- Successful alcohol cessation defined as zero alcohol intake at 6 weeks of intervention/end of intervention and surgery and below risky limits (<14 units per week) at 30 days, 3 months, and 6 months postoperatively. Validation by blood phosphatidylethanol (B-Peth) and urine ethylglucuronide (U-EtG)
- Physical activity: at least 3.5 hours every week [45] throughout the study period

Any improvement is defined as a reduction of smoking or alcohol intake, any loss of body fat mass, any improvement in risk of malnutrition (including NRS score or albumin level [3]) or physical activity. Quit rates for the separate lifestyles will be descriptively reported.

In the intervention group, the association between motivation, priority, and self-efficacy, respectively, and the actual change in lifestyles 6 months postoperative will be examined. The participants’ self-estimated motivation, priority and self-efficacy, respectively, for lifestyle changes are collected prior to the intervention. The information is measured on a scale from 0–10 (10 being the highest level) for each participant in each of the three areas using the line tool (line 1: importance of avoiding complications; line 2: importance of changing lifestyle now; line 3: self-efficacy of actually changing lifestyle), Fig 3. Another outcome is themes in participants’ reflections on advantages and disadvantages of the intervention. In the intervention group, the participants will use the box tool to describe their reflections on the advantages and disadvantages of the lifestyle intervention prior to beginning the intervention. Themes will be derived through qualitative analysis. Finally, associations between meeting adherence and change in risky lifestyle in the intervention group will be explored. Meeting adherence is defined as attendance at meetings and measured as a proportion. Completion is defined as attending ≥75% of meetings.

Other pre-specified and descriptively reported outcomes are the number and proportion of participants with an improvement in ASA score level compared with baseline and related to an improvement of one or more risky lifestyles, and the number and proportion of participants with improvement in frailty score compared to baseline. Both outcomes will be evaluated at the end of intervention, at surgery, after 30 days, three months, and six months of follow-up. Frailty is measured by Fried’s Modified Frailty Score [57]. This frailty scale is chosen as it is associated with the development of complications [57].

#### Health.

Health-related quality of life (HrQoL) will be measured by the European Quality of life 5 Dimensions (EQ-5D) instrument [58] in a Danish translated validated version [59], a generic and well-used instrument in many clinical studies. It includes the five dimensions of mobility, self-care, usual activities, pain/discomfort, and anxiety/depression, and each is divided into five levels of perceived difficulties. The higher the level, the more challenges are perceived by the participant. The overall health will be evaluated with a visual analog scale going from 0 to 100 with a higher score representing better health. Health-related quality of life will be assessed at baseline and then again at end of intervention, at surgery, and at follow-up 30 days, three months, and six months after surgery. Outcomes will be calculated as the number and proportion of participants with improvement of HrQoL compared with baseline at the different time points.

Morbidity after two years is measured as grouped diagnoses groups via the International Classification of Diseases 10^th^ revision (ICD-10) Danish version [60] based on data from the Danish National Patient Registry [61] and will be descriptively reported.

Through qualitative analysis of transcribed semi-structured interviews, themes related to the participants’ reflections on the STRONG programme, their health status, changing lifestyle, and participating in the STRONG-Hernia-trial will be derived and reported.

#### Costs.

Costs will be reported after 30 days, six months, and two years postoperatively as a comparison between intervention and control group. Costs will be measured as individual direct and indirect healthcare costs per participant based on perioperative costs, time back to work or reuptake of previous activities, total stay, and visits/contacts to hospital and primary healthcare (from the national registries).

Cost-effectiveness based on data up to two years after surgery will also be reported. In addition to the costs mentioned above, data includes the EQ-5D as previously described, morbidity after two years measured as described above, visits and stay at hospital (via the Danish National Patient Registry [61]), visits in primary care (via the primary care registry, Danish National Health Service Registry [62]), prescribed medicine (via the Medicine Rregistry [63]), and mortality after two years (via the Danish Civil Registration System [64]).

### Participant timeline

Participants will be enrolled, randomised to the intervention or control group, and begin the trial around the same time (preferably on the same day if possible). The intervention group will meet with project staff once a week, approximately, for a total of six meetings, Figs 1 and 2. Both groups will have a follow-up meeting at the time of surgery and after one, three, and six months. If more than eight weeks pass between inclusion and surgery, the control group will have an extra meeting after six weeks corresponding to the end of intervention meeting (the sixth meeting) in the intervention group.

### Sample size

A total of 400 participants will be included. The power calculation is based on the literature presented in the introduction section showing that postoperative complications, in general, can be halved after intensive programmes for smoking, alcohol, and malnutrition [10–14]. The impact of physical activity is lower, and obesity is sparsely investigated but targeting co-existing SNAP factors like the STRONG programme should increase the effect due to positive interactions. Therefore, the main hypothesis is clinically relevant.

Postoperative complication rates after ventral hernia repair and the definitions vary (e.g., surgical site infections or respiratory complications, exclusively), and often different ventral hernias and different surgical techniques are mixed as one population in the literature (e.g., primary and secondary repairs, umbilical and incisional hernias, and mesh and non-mesh repairs). An RCT and a register-based study on primary ventral hernia repairs with mesh found similar complication rates (broad definition) of around 25% [65,66]. A cohort study has shown that reoperation rates underestimate the overall risk of recurrence by four- to fivefold and reported a combined reoperation and hernia recurrence rate of around 15% [67]. Furthermore, surgical site occurrences are common after both incisional and primary ventral hernia repairs [68,69]. In this trial, we include all complications requiring any treatment (including complications handled outside the hospitals such as medical pain prescriptions for prolonged pain and antibiotics by general practitioners), hence, a higher complication rate could be expected. Finally, postoperative complications are more common in patients with risky lifestyles such as smoking, alcohol use disorder, and obesity [4,68,70], so we expect higher complication rates in the trial population than in the general ventral hernia repair population, which consists of a mixture of patients with and without risky lifestyles. For ventral hernia in participants with risky lifestyles, STRONG would expectantly reduce by half the overall rate of complications requiring treatment from conservatively 20% to 10%. When using 80% power and 2 × alpha = 0.05, the number of participants needed is 2 × 199 participants.

### Recruitment

The participant flow at the three included departments should be high enough to achieve the target sample size for which the primary outcome is powered. If necessary, more departments – including in the private sector – may be included after approval from the Ethical Scientific Committee and the Danish Data Protection Agency.

Recruitment takes place in connection with outpatient visits where participants are already in the hospital. Intervention and follow-ups are planned together with participants to accommodate their wishes within the framework of the study.

### Assignment of interventions

#### Allocation.

The allocation sequence will be generated with computer-generated random numbers. The randomisation is stratified by centre, number of risky SNAP factors, and hernia type. Participants are allocated in a 1:1 ratio in varying block sizes from 2–6. The participants are randomised and allocated directly after giving informed consent by the project personnel enrolling them. They use a computerised randomisation system in research electronic data capture (REDCap) with the randomisation scheme hidden from all project personnel. The system is always accessible, and the use is logged. The allocation sequence as described above will be generated and uploaded to REDCap by a person not otherwise involved in the trial.

#### Blinding.

Blinding intensive lifestyle intervention is rarely achievable. Allocation of the participants is not registered in the medical record system, but there is no assurance that participants will not disclose their intervention group allocation to clinicians and outcome assessors. However, all biomarker analyses for validation and all statistical analyses will be conducted blinded.

#### Data collection and management.

Plans for assessment and collection of outcomes, baseline, and other trial data are illustrated in Table 1. At baseline, participants will be asked an elaborate set of questions regarding their risky SNAP factors identified at screening. Smokers will be asked questions to establish their smoking pattern, nicotine dependency, Fagerström’s test for nicotine dependency [51], and previous experiences with smoking cessation [71]. For participants who are obese and/or at risk of malnutrition data will be collected regarding their nutritional intake [72], NRS score [43], and use of weight loss medication. Waist measurement and bioimpendance measurements will also be collected. The bioimpedance measurements at baseline include weight in kg, BMI, muscle mass, body fat mass, and total body water. Collected data for risky drinkers include the number of drinks per week (using the timeline follow-back method), AUDIT-C score [73], DSM5 score [74], and if the participant experiences withdrawal symptoms then also a CIWA-AR score [75]. The level of physical activity will be explored qualitatively equal to the screening. Most of the data items collected for risky drinkers, participants who are obese/at risk of malnutrition, and participants who are physically inactive at baseline will also be collected again at different meetings in the intervention group, and for all at the time point of the end of intervention/surgery, after 30 days, 90 days and six months postoperatively, see Table 1.

**Table 1:** Overview of collected data items with time points.

Data variable	Definition & instrument	Inclusion	Intervention group only							Surgery	Postoperatively			
			0w	1w	2w	3w	4w	5w	5w		1m	3m	6m	2y
**Screening**														
Smoking daily	≥1 gram (y/n)	X							X	X	X	X	X	
Obesity	BMI > 30 kg/m^2^ (y/n)	X							X	X	X	X	X	
Risk of malnutrition	NRS ≥ 3 (y/n) [43]	X							X	X	X	X	X	
Alcohol use	>14 units/w (y/n)	X							X	X	X	X	X	
Physical inactivity	<3.5 hours/w (y/n)	X							X	X	X	X	X	
**Demographics**														
Age	In years	X												
Sex	Men/women	X												
Housing	Living alone & type	X												
Education level	8 categories	X												
Socioeconomics	16 categories	X												
Charlson Comorbidity Score	Medical records [76]	X												
**Surgical data**														
Complications	Medical records + interview + clinical examination										X	X	X	
Clavien-Dindo	Medical records + interview [77]										X	X	X	
CCI	Medical records + interview [55]										X	X	X	
Hernia recurrence	Clinical examination										X	X	X	
Reop. for hernia recurrence	Medical records										X	X	X	
Length of stay	Medical records										X			
Readmission	Medical records											X		
Return to work/usual activities	Interview										X	X	X	
Primary care visits	Interview										X			
Intraoperative data	Medical records									X				
**Lifestyle data**														
Smoking history	Interview [71]	X												
Smoking status	Interview ± CO test	X		X	X	X	X		X	X	X	X	X	
Nutritional intake	Interview	X												
Nutrition status	Interview + measurements	X			X				X	X	X	X	X	
Alcohol intake	TLFB	X		X	X	X	X		X	X	X	X	X	
Alcohol misuse/dependency	DSM5-score [74], AUDIT-C [73]	X			X				X	X	X	X	X	
Alcohol withdrawal symptoms	CIWA-AR [75]	X		X	X	X	X		X	X	X	X	X	
Alcohol validation	Breath test	X		X	X	X	X		X	X	X	X	X	
Physical activity	Interview			X	X	X	X		X	X	X	X	X	
ASA score	Medical records + interview [78]	X			X				X	X	X	X	X	
Frailty score	Measurements + interview [57]	X			X				X	X	X	X	X	
**Health data**														
HrQoL	ED-5Q [58]	X			X				X	X	X	X	X	
Morbidity	Register-based [61]													X
Patient reflections	Semi-structured interview	X												
**Cost data**														
Healthcare costs	Register-based [61–64]										X		X	X
**Intervention specific data**														
Participant expectations	The line tool [46]		X											
Participant reflections	The box tool [47]		X											
Healthy SNAP days	Logbook + interview ± measurements			X	X	X	X	X						
Harms	Interview + medical records			X	X	X	X	X						
Compliance	Interview			X	X	X	X	X		X				
**Laboratory tests**														
Routine blood tests	Hgb, albumin, ASAT, bilirubin	X			X				X	X	X	X	X	
Smoking validation	Urine cotinine	X			X				X	X	X	X	X	
Alcohol validation	B-Peth, U-EtG	X			X				X	X	X	X	X	

Semi-structured interviews are conducted according to a semi-structured interview-guide. Interviews are structured questions orally discussed with the participants.

ASAT: aspartate aminotransferase, BMI: body mass index, B-PEth: blood phosphatidyl-ethanol, CCI: Comprehensive Complication Index, hgb: hemoglobin, m: month(s), n: no, NRS: nutritional risk score, reop: reoperation, U-Etg: urine ethyl glucuronide, w: week, y: yes.

At each meeting during the intervention, participants in the intervention group are interviewed about healthy days, i.e., days without smoking or alcohol intake, eating according to the nutritional plan and being physically active according to the plan as well as use of any prescribed support medication.

#### Plans to promote participant retention and complete follow-up.

All meetings are planned with the participants to accommodate their wishes best possible within the framework of the study. The Ethical Scientific Committee has approved that drop-out participants are asked for consent to follow them in the medical record system and a telephone interview. It is our experience from previous studies that 90% of drop-out participants accept this. Therefore, the primary outcome, complications requiring treatment, will be available for close to all randomised participants.

#### Data management and confidentiality.

REDCap is used for safe storage of the gathered data. It is a secure platform approved by the Danish Data Protection Agency, Capital Region of Denmark. Only the project group has access and all access is logged. Throughout the trial, the conduction follows the Danish Data Protection Agency guidelines. After the study period has ended, all personal information will be destroyed and the other data will be stored at the Danish National Data Archive in line with the Danish and European guidelines for data management and protection.

#### Biological specimens for genetic or molecular analysis in this trial/future use.

All participants have blood and urine tests sampled at baseline and the four scheduled follow-ups (surgery, 30 days, 90 days, and six months) for the identification of alcohol- and tobacco biomarkers (B-PetH, U-EtG and U-cotinine), for routine nutrition profile analyses (plasma-albumin), and routine analyses on blood-hemoglobin, plasma aspartate transaminase, and plasma-bilirubin as well as for measurement of possible new markers developed during the project period. In total, approximately 30 ml blood and 10 ml urine are collected at inclusion, end of intervention (only if it is more than a week before surgery), surgery, and at the three follow-ups (one, three, and six months). The intervention group will also have blood and urine colleced at the third intervention meeting. If additional informed consent is given, half of the samples are kept for analyses of new biomarkers possibly developed during the project period (see below).

The routine analyses are performed at the local centres. The analyses of the established biomarkers on tobacco and alcohol are performed together for one factor at a time, mainly after collection of all samples with the aim of validating the self-reported data on lifestyle outcomes. During the project and until analysed, they will be stored in a biobank at the Parker laboratory at Bispebjerg-Frederiksberg Hospital.

New markers may be developed during the project period, and we will therefore (if additional informed consent is given) keep half of the samples in a pseudo-anonymised form stored for further analyses related to measurement of new markers for risk reduction at surgery developed during the study period (until December 31 2033). The samples can be used in future research only after approval from the Scientific Ethical Committee and the Danish Data Protection Agency.

### Statistical methods

Analyses are conducted as Intention-to-Treat. They are done blinded, thus only the statistician will have access to the final dataset and not the investigators. We will use Fisher’s exact test or Chi square test for frequencies (depending on the expected values), Mann-Whitney (non-parametric) for continuous unpaired variables, and p < 0.05 is chosen for statistical significance. To benefit future meta-analyses, we will also give the parametric results. This would also meet the tradition that costs are usually reported with means and standard error (SE), mean difference (MD), and 95% confidence interval (CI). Bootstrapping procedures are used to calculate the cost-effectiveness plane and the acceptability curve by repeatedly resampling the data (1,000 incremental cost and effect pairs). Descriptive outcome data will be reported with numbers, proportions (%), and 95% CI.

Sensitivity analyses will be performed for differences in local standards for the control groups among the surgical centres. Analyses as per-protocol, dose-response, prediction, sensitivity, and the other secondary analyses are done by logistic regression models including control for confounders and effect-modifiers and reported as odds ratios with 95% CIs which are considered significant if they do not involve 1.00.

Absolute risk reduction (ARR) is calculated as the events in the control group minus the events in the intervention group, and the relative risk reduction as the ARR divided by the events in the control group. The number needed to treat was calculated by 1/ARR. We will present the mortality as Kaplan-Meier plots and test by the log rank test. In case of missing health data, the lifestyle at baseline will be imputed as the results in order not to overestimate the results of changing lifestyle. This would expectantly impact the results to a similar degree in both groups.

Although the assumptions for the sample size calculation are conservative, they are associated with uncertainties. Furthermore, as this is the first RCT to be conducted with this intensive and broad prehabilitation intervention in minor surgery, a blinded interim data assessment will be conducted to assess whether the assumptions hold.

The qualitative analyses are done using Kirsti Malterud’s approach of systematic text condensation [79]. After import into NVivo® qualitative data analysis software version 11, the systematic text condensation in the four steps takes place: 1) Total impression of all answers and identifying preliminary themes, 2) coding by identifying and sorting meaning units, 3) condensation into code groups, and 4) synthesising the condensates into a story grounded in the empirical data.

A more detailed statistical analysis plan (SAP) will be prepared and locked before any data analyses are started.

### Oversight and monitoring

The project is managed daily and coordinated through close collaboration between the STRONG team and the involved departments of surgery. Participant panel meetings take place twice a year. The trial steering committee, which includes the principal investigator and local project leaders who oversee the funding, meets two to four times annually. The project management group holds monthly meetings. Additional meetings are arranged as needed.

Daily data monitoring is conducted by the STRONG team while an external independent researcher reviews it biannually. Data regarding any adverse events or other unintended effects related to the intervention are gathered during each meeting. In addition, the participants report on the hotline (telephone and mail). If such events occur, they will be reported to the Danish Medicines Agency. Furthermore, participants have the option to contact the staff through the provided hotline.

### Ethics and dissemination

The trial is approved by the Danish Scientific Ethical Committee (protocol version 5, latest approval date 21^th^ of May 2024, H-23028872) and the Danish Data Protection Agency (P-2023–14284). Minor changes were made between the different versions of the protocol including clarifications of included type of ventral hernias and addition of a planned sub-study estimating prevalence of SNAP factors in the potential study population. If any further important protocol modifications are made, they will be communicated to the involved parties, the Danish Scientific Ethical Committee, and the Danish Data Protection Agency. We will also update the trial registry at clinicaltrials.gov as soon as possible.

#### Consent.

Participants will be approached by project personnel in person or by telephone in connection with the outpatient meeting and initial planning of surgery. Here, they will be screened for eligibility and eligible participants will be invited to participate in the trial. A project person will give oral and written information and answer questions. Written, informed consent for participating in the trial will be collected face-to-face for all participants upon inclusion and in time to allow enough time for prehabilitation (Fig 1).

Since new biomarkers may be developed during the trial period, participants will be asked for additional written, informed consent for keeping half of the blood and urine samples stored in a biobank for future research for potential further analyses related to measurements of potential new markers for risk reduction at surgery during the study period.

To investigate if eligible participants who decline to participate in the trial differ from those enrolled, we will ask for permission to follow them per- and postoperatively via the medical records (up to 6 months postoperatively) and for 2 years in the health registries. Those who accept are asked to provide informed consent.

#### Provisions for post-trial care.

Participants in Danish investigator-initiated trials will be protected by the Danish Act on the Right to Complain and Receive Compensation within the Danish Health Service (Patientforsikringsloven). This act specifically applies to patients receiving treatment in Danish public hospitals and is a standard practice for such trials in Denmark.

#### Dissemination plans.

We plan to publish all results when the trial is finalised. Both positive, negative, and inconclusive results will be sought to be published in scientific journals. Additionally, results will be communicated to the public through lectures as well as clinical, research, and public networks.

### Trial status

Recruitment began March 4, 2024, and is ongoing. Participant recruitment is expected to be completed in 2.5 years. Data collection will be completed 6 months after last participant has been recruited, i.e., around March 2027. Results are expected to be analyzed within six months after last participant follow-up, thus September 2027. Current trial status will be updated continuously on clinicaltrials.gov.

## Discussion

This RCT investigates the effect of intensive combined interventions for up to five risky lifestyles (smoking, malnutrition, obesity, risky alcohol intake, and physical inactivity) before the minor surgical procedure of ventral hernia repair. For smoking, the relatively small effect on public health of brief and even very brief lifestyle interventions are cost-effective at a population level and therefore important for society at longer term [80]. However, in surgical settings, the small improvements after brief interventions have not been shown to affect postoperative complications [10]. The GSP [40] has proven very effective with quit rates of around 50% for smoking and alcohol on short-term following the intensive interventions and a similar reduction in postoperative complications when intensive interventions are used prior to surgery [10,11]. Therefore, the STRONG programme is based on the GSP programme and uses intensive rather than brief interventions [81].

The combined programme is designed for individual delivery by one trained nurse as a part of the surgical pathway. This approach is based on previous experiences showing feasibility problems of several separate interventions delivered by separate personnel, increased administrative resources, and participant preferences toward one integrated programme. A pilot programme combining interventions for alcohol/drug addiction participants found that integrating multiple interventions into one session and delivered by one person was more feasible and aligned with participant perspectives [82]. This combined approach has been well-received in a recent intervention trial on perioperative smoking and alcohol cessation [83]. If the STRONG programme proves effective, it will be easier to implement into practice than a fragmented programme involving different personnel.

An intensive combined programme may be time-consuming for the participant. As ventral hernia repair may not be as life-altering as getting a cancer diagnosis and undergoing chemotherapy while awaiting major surgery (as in the STRONG-Cancer trial [84]), it may be more difficult and important to motivate participants and make them see the advantage of participating in this trial. Therefore, we expect a learning curve in inclusion rates.

This study will bring more knowledge on the effect of prehabilitation. Until now, most prehabilitation research is related to major/cancer surgery. If this trial shows positive results in participants undergoing minor surgery, it will expand the area of use and interest for prehabilitation significantly.

## Supporting information

S1 File SPIRIT 2013 checklist. (PDF)

S2 File Protocol approved by ethics committee. (PDF)

## Abbreviations

ASA: American Society of Anaesthesiologists
BMI: body mass index
CCI: Comprehensive Complication Index
CO: carbon monoxide
EQ-5D: European Quality of life 5 Dimensions
GSP: gold standard programmeme
HrQoL: health-related quality of life
ICD-10: International Classification of Diseases 10th revision
IMT: inspiratory muscle training
LoS: length of stay
NRS: nutritional risk score
P-PEth: plasma phosphatidylethanol
RCT: Randomised controlled trial
REDCap: research electronic data capture
SNAP: smoking, nutrition (malnutrition and obesity), alcohol, physical inactivity
U-cotinine: Urine cotinine
U-EtG: urine ethylglucuronide

## References

[1] Grønkjær M, Eliasen M, Skov-Ettrup LS, Tolstrup JS, Christiansen AH, Mikkelsen SS, et al. Preoperative smoking status and postoperative complications: a systematic review and meta-analysis. Ann Surg. 2014;259(1):52–71. doi: 10.1097/SLA.0b013e3182911913 23799418

[2] Eliasen M, Grønkjær M, Skov-Ettrup LS, Mikkelsen SS, Becker U, Tolstrup JS, et al. Preoperative alcohol consumption and postoperative complications: a systematic review and meta-analysis. Ann Surg. 2013;258(6):930–42. doi: 10.1097/SLA.0b013e3182988d59 23732268

[3] Weimann A, Braga M, Carli F, Higashiguchi T, Hübner M, Klek S. Espen practical guideline: clinical nutrition in surgery. Clin Nutr. 2021;40:4745–61. 34242915 10.1016/j.clnu.2021.03.031

[4] Park H, de Virgilio C, Kim DY, Shover AL, Moazzez A. Effects of smoking and different BMI cutoff points on surgical site infection after elective open ventral hernia repair. Hernia. 2021;25(2):337–43. doi: 10.1007/s10029-020-02190-x 32318887

[5] Bohlin KS, Ankardal M, Stjerndahl J-H, Lindkvist H, Milsom I. Influence of the modifiable life-style factors body mass index and smoking on the outcome of hysterectomy. Acta Obstet Gynecol Scand. 2016;95(1):65–73. doi: 10.1111/aogs.12794 26459279

[6] Snowden CP, Prentis J, Jacques B, Anderson H, Manas D, Jones D, et al. Cardiorespiratory fitness predicts mortality and hospital length of stay after major elective surgery in older people. Ann Surg. 2013;257(6):999–1004. doi: 10.1097/SLA.0b013e31828dbac2 23665968

[7] Onerup A, Angenete E, Bonfre P, Börjesson M, Haglind E, Wessman C. Self-assessed preoperative level of habitual physical activity predicted postoperative complications after colorectal cancer surgery: a prospective observational cohort study. Eur J Surg Oncol. 2019;45:2045–51. 31217078 10.1016/j.ejso.2019.06.019

[8] Kehlet H. Enhanced postoperative recovery: good from afar, but far from good?. Anaesthesia. 2020;75 Suppl 1:e54–61. doi: 10.1111/anae.14860 31903577

[9] Harryman C, Plymale MA, Stearns E, Davenport DL, Chang W, Roth JS. Enhanced value with implementation of an ERAS protocol for ventral hernia repair. Surg Endosc. 2020;34(9):3949–55. doi: 10.1007/s00464-019-07166-2 31576444

[10] Thomsen T, Villebro N, Møller AM. Interventions for preoperative smoking cessation. Cochrane Database Syst Rev. 2014;2014(3):CD002294. doi: 10.1002/14651858.CD002294.pub4 24671929 PMC7138216

[11] Egholm JW, Pedersen B, Møller AM, Adami J, Juhl CB, Tønnesen H. Perioperative alcohol cessation intervention for postoperative complications. Cochrane Database Syst Rev. 2018;11(11):CD008343. doi: 10.1002/14651858.CD008343.pub3 30408162 PMC6517044

[12] Burden S, Todd C, Hill J, Lal S. Pre-operative nutrition support in patients undergoing gastrointestinal surgery. Cochrane Database Syst Rev. 2012;11:CD008879. doi: 10.1002/14651858.CD008879.pub2 23152265

[13] Jie B, Jiang Z-M, Nolan MT, Zhu S-N, Yu K, Kondrup J. Impact of preoperative nutritional support on clinical outcome in abdominal surgical patients at nutritional risk. Nutrition. 2012;28(10):1022–7. doi: 10.1016/j.nut.2012.01.017 22673593

[14] Weimann A, Braga M, Carli F, Higashiguchi T, Hübner M, Klek S, et al. ESPEN guideline: Clinical nutrition in surgery. Clin Nutr. 2017;36(3):623–50. doi: 10.1016/j.clnu.2017.02.013 28385477

[15] Smith NA, Martin G, Marginson B. Preoperative assessment and prehabilitation in patients with obesity undergoing non-bariatric surgery: A systematic review. J Clin Anesth. 2022;78:110676. doi: 10.1016/j.jclinane.2022.110676 35152081

[16] Duro-Ocana P, Zambolin F, Jones AW, Bryan A, Moore J, Quraishi-Akhtar T, et al. Efficacy of supervised exercise prehabilitation programs to improve major abdominal surgery outcomes: A systematic review and meta-analysis. J Clin Anesth. 2023;86:111053. doi: 10.1016/j.jclinane.2023.111053 36736208

[17] Molenaar CJ, van Rooijen SJ, Fokkenrood HJ, Roumen RM, Janssen L, Slooter GD. Prehabilitation versus no prehabilitation to improve functional capacity, reduce postoperative complications and improve quality of life in colorectal cancer surgery. Cochrane Database Syst Rev. 2023;5(5):CD013259. doi: 10.1002/14651858.CD013259.pub3 37162250 PMC10171468

[18] Bausys A, Luksta M, Anglickiene G, Maneikiene VV, Kryzauskas M, Rybakovas A, et al. Effect of home-based prehabilitation on postoperative complications after surgery for gastric cancer: randomized clinical trial. Br J Surg. 2023;110(12):1800–7. doi: 10.1093/bjs/znad312 37750588

[19] McIsaac DI, Gill M, Boland L, Hutton B, Branje K, Shaw J, et al. Prehabilitation in adult patients undergoing surgery: an umbrella review of systematic reviews. Br J Anaesth. 2022;128(2):244–57. doi: 10.1016/j.bja.2021.11.014 34922735

[20] Oppedal K, Nesvåg S, Pedersen B, Skjøtskift S, Aarstad AKH, Ullaland S, et al. Health and the need for health promotion in hospital patients. Eur J Public Health. 2011;21(6):744–9. doi: 10.1093/eurpub/ckq148 20943993

[21] van Rooijen S, Carli F, Dalton SO, Johansen C, Dieleman J, Roumen R, et al. Preoperative modifiable risk factors in colorectal surgery: an observational cohort study identifying the possible value of prehabilitation. Acta Oncol. 2017;56(2):329–34. doi: 10.1080/0284186X.2016.1267872 28067102

[22] Lydom LN, Jensen SAMS, Lauridsen SV, Rasmussen M, Christensen R, Joensen UN, et al. Perioperative combined lifestyle interventions for smoking, malnutrition, obesity, alcohol drinking and physical activity: impact on postoperative complications - a systematic review and network meta-analysis. PROSPERO; 2022 [cited February 25, 2025]. https://www.crd.york.ac.uk/prospero/display_record.php?ID=CRD42022282611 10.12688/f1000research.150880.2 PMC12198731 40574788

[23] Poulose BK, Shelton J, Phillips S, Moore D, Nealon W, Penson D, et al. Epidemiology and cost of ventral hernia repair: making the case for hernia research. Hernia. 2012;16(2):179–83. doi: 10.1007/s10029-011-0879-9 21904861

[24] Kokotovic D, Sjølander H, Gögenur I, Helgstrand F. Watchful waiting as a treatment strategy for patients with a ventral hernia appears to be safe. Hernia. 2016;20(2):281–7. doi: 10.1007/s10029-016-1464-z 26838293

[25] Jolissaint JS, Dieffenbach BV, Tsai TC, Pernar LI, Shoji BT, Ashley SW, et al. Surgical site occurrences, not body mass index, increase the long-term risk of ventral hernia recurrence. Surgery. 2020;167(4):765–71. doi: 10.1016/j.surg.2020.01.001 32063341 PMC8186954

[26] Ayuso SA, Robinson JN, Colavita PD, Heniford BT. Smoking, Obesity, and the Elective Operation. Surg Clin North Am. 2021;101(6):981–93. doi: 10.1016/j.suc.2021.05.025 34774276

[27] Jensen KK, East B, Jisova B, Cano ML, Cavallaro G, Jørgensen LN, et al. The European Hernia Society prehabilitation project: a systematic review of patient prehabilitation prior to ventral hernia surgery. Hernia. 2022;26:715–26. 35212807 10.1007/s10029-022-02573-2

[28] Lindström D, Sadr Azodi O, Wladis A, Tønnesen H, Linder S, Nåsell H, et al. Effects of a perioperative smoking cessation intervention on postoperative complications: a randomized trial. Ann Surg. 2008;248(5):739–45. doi: 10.1097/SLA.0b013e3181889d0d 18948800

[29] Sørensen LT, Hemmingsen U, Jørgensen T. Strategies of smoking cessation intervention before hernia surgery--effect on perioperative smoking behavior. Hernia. 2007;11(4):327–33. doi: 10.1007/s10029-007-0229-0 17503161

[30] Liang MK, Bernardi K, Holihan JL, Cherla DV, Escamilla R, Lew DF, et al. Modifying Risks in Ventral Hernia Patients With Prehabilitation: A Randomized Controlled Trial. Ann Surg. 2018;268(4):674–80. doi: 10.1097/SLA.0000000000002961 30048306

[31] Bernardi K, Olavarria OA, Dhanani NH, Lyons N, Holihan JL, Cherla DV, et al. Two-year Outcomes of Prehabilitation Among Obese Patients With Ventral Hernias: A Randomized Controlled Trial (NCT02365194). Ann Surg. 2022;275(2):288–94. doi: 10.1097/SLA.0000000000004486 33201119

[32] Pekkarinen T, Mustajoki P. Use of very low-calorie diet in preoperative weight loss: efficacy and safety. Obes Res. 1997;5(6):595–602. doi: 10.1002/j.1550-8528.1997.tb00581.x 9449145

[33] Goldberg R, Parker M, Stauffer J, Moti S, Sylvia J, Ames G. Surgeon’s requirement for obesity reduction: its influence on weight loss. Am Surg. 2012;78:325–8. 22524771

[34] Eid GM, Wikiel KJ, Entabi F, Saleem M. Ventral hernias in morbidly obese patients: a suggested algorithm for operative repair. Obes Surg. 2013;23(5):703–9. doi: 10.1007/s11695-013-0883-5 23494458

[35] Plymale MA, Davenport DL, Roth JS. Outcomes Experienced by Patients Presenting with Ventral Hernia and Morbid Obesity in a Surgical Clinic. Am Surg. 2017;83(8):e344-346. doi: 10.1177/000313481708300828 28822383

[36] Rosen MJ, Aydogdu K, Grafmiller K, Petro CC, Faiman GH, Prabhu A. A Multidisciplinary Approach to Medical Weight Loss Prior to Complex Abdominal Wall Reconstruction: Is it Feasible?. J Gastrointest Surg. 2015;19(8):1399–406. doi: 10.1007/s11605-015-2856-6 26001369

[37] Carli F, Zavorsky GS. Optimizing functional exercise capacity in the elderly surgical population. Curr Opin Clin Nutr Metab Care. 2005;8(1):23–32. doi: 10.1097/00075197-200501000-00005 15585997

[38] Chabot K, Gillis C, Carli F. Prehabilitation: metabolic considerations. Curr Opin Clin Nutr Metab Care. 2020;23(4):271–6. doi: 10.1097/MCO.0000000000000663 32398440

[39] Tønnesen H. Engage in the process of change; facts and methods. Tønnesen H, editor. Publications of WHO Regional Office for Europe; 2012.

[40] Rasmussen M, Fernández E, Tønnesen H. Effectiveness of the Gold Standard Programme compared with other smoking cessation interventions in Denmark: a cohort study. BMJ Open. 2017;7(2):e013553. doi: 10.1136/bmjopen-2016-013553 28242770 PMC5337720

[41] Disha SR, Merin Eldhose K, Shetty Y. Exploring the trend of use of qualitative methods in randomized controlled trials. Perspect Clin Res. 2023;14(4):207–8. doi: 10.4103/picr.picr_131_22 38025293 PMC10679569

[42] Chan A, Tetzlaff J, Gøtzsche P, Altman D, Mann H, Berlin J. Spirit 2013 explanation and elaboration: guidance for protocols of clinical trials. BMJ. 2013;346:e7586. 10.1136/bmj.e7586 PMC3541470 23303884

[43] Kondrup J, Rasmussen HH, Hamberg O, Stanga Z, Ad Hoc ESPEN Working Group. Nutritional risk screening (NRS 2002): a new method based on an analysis of controlled clinical trials. Clin Nutr. 2003;22(3):321–36. doi: 10.1016/s0261-5614(02)00214-5 12765673

[44] Rubinsky AD, Bishop MJ, Maynard C, Henderson WG, Hawn MT, Harris AHS, et al. Postoperative risks associated with alcohol screening depend on documented drinking at the time of surgery. Drug Alcohol Depend. 2013;132(3):521–7. doi: 10.1016/j.drugalcdep.2013.03.022 23683792

[45] Aadahl M, Jørgensen T. Validation of a new self-report instrument for measuring physical activity. Med Sci Sports Exerc. 2003;35(7):1196–202. doi: 10.1249/01.MSS.0000074446.02192.14 12840642

[46] Miller W, Rollnick S. Motivational interviewing, preparing people to change addictive behaviour. 2002.

[47] Janis I, Mann L. Decision making: a psychological analysis of conflict, choice, and commitment. 1977.

[48] Prochaska JO, DiClemente CC, Norcross JC. In search of how people change. Applications to addictive behaviors. Am Psychol. 1992;47(9):1102–14. doi: 10.1037//0003-066x.47.9.1102 1329589

[49] Lauridsen SV, Thomsen T, Jensen JB, Kallemose T, Schmidt Behrend M, Steffensen K, et al. Effect of a Smoking and Alcohol Cessation Intervention Initiated Shortly Before Radical Cystectomy-the STOP-OP Study: A Randomised Clinical Trial. Eur Urol Focus. 2022;8(6):1650–8. doi: 10.1016/j.euf.2022.02.005 35241394

[50] Egholm JWM, Pedersen B, Oppedal K, Madsen BL, Lauritzen JB, Rasmussen M, et al. Minor effect of patient education for alcohol cessation intervention on outcomes after acute fracture surgery: a randomized trial of 70 patients. Acta Orthop. 2022;93:424–31. doi: 10.2340/17453674.2022.2482 35417027 PMC9006589

[51] Heatherton TF, Kozlowski LT, Frecker RC, Fagerstrom KO. The fagerstrom test for nicotine dependence: a revision of the fagerstrom tolerance questionnaire. Br J Addict. 1991;86:1119–27. 1932883 10.1111/j.1360-0443.1991.tb01879.x

[52] Grimble RF. Basics in clinical nutrition: immunonutrition - nutrients which influence immunity: effect and mechanism of action. Clin Nutr ESPEN. 2009;4:e10–3.

[53] Fleurent-Grégoire C, Burgess N, Denehy L, Edbrooke L, Engel D, Testa GD. Outcomes reported in randomised trials of surgical prehabilitation: a scoping review. Br J Anaesth. 2024;133:42–57. 38570300 10.1016/j.bja.2024.01.046 PMC11213997

[54] Dindo D, Demartines N, Clavien P-A. Classification of surgical complications: a new proposal with evaluation in a cohort of 6336 patients and results of a survey. Ann Surg. 2004;240(2):205–13. doi: 10.1097/01.sla.0000133083.54934.ae 15273542 PMC1360123

[55] Slankamenac K, Graf R, Barkun J, Puhan MA, Clavien P-A. The comprehensive complication index: a novel continuous scale to measure surgical morbidity. Ann Surg. 2013;258(1):1–7. doi: 10.1097/SLA.0b013e318296c732 23728278

[56] Sjælland REiRHoR. Overvægt/fedme (adipositas) - BMI 25 eller højere, rådgivning og behandling af voksne patienter (16 år og derover). VIP; 2017.

[57] Birkelbach O, Mörgeli R, Spies C, Olbert M, Weiss B, Brauner M, et al. Routine frailty assessment predicts postoperative complications in elderly patients across surgical disciplines - a retrospective observational study. BMC Anesthesiol. 2019;19(1):204. doi: 10.1186/s12871-019-0880-x 31699033 PMC6839249

[58] Stolk E, Ludwig K, Rand K, van Hout B, Ramos-Goñi JM. Overview, Update, and Lessons Learned From the International EQ-5D-5L Valuation Work: Version 2 of the EQ-5D-5L Valuation Protocol. Value Health. 2019;22(1):23–30. doi: 10.1016/j.jval.2018.05.010 30661630

[59] Jensen MB, Jensen CE, Gudex C, Pedersen KM, Sørensen SS, Ehlers LH. Danish population health measured by the EQ-5D-5L. Scand J Public Health. 2023;51(2):241–9. doi: 10.1177/14034948211058060 34847818 PMC9969307

[60] The Danish Health Data Authority: SKS-browser vers 4.06. n.d. [cited February 25, 2025].https://medinfo.dk/sks/brows.php

[61] Schmidt M, Schmidt SAJ, Sandegaard JL, Ehrenstein V, Pedersen L, Sørensen HT. The Danish National Patient Registry: a review of content, data quality, and research potential. Clin Epidemiol. 2015;7:449–90. doi: 10.2147/CLEP.S91125 26604824 PMC4655913

[62] Sahl Andersen J, De Fine Olivarius N, Krasnik A. The Danish national health service register. Scand J Public Health. 2011;39:34–7. 21775348 10.1177/1403494810394718

[63] Wallach Kildemoes H, Toft Sørensen H, Hallas J. The Danish national prescription registry. Scand J Public Health. 2011;39:38–41. 21775349 10.1177/1403494810394717

[64] Schmidt M, Pedersen L, Sørensen H. The Danish civil registration system as a tool in epidemiology. Eur J Epidemiol. 2014;29:541–9. 24965263 10.1007/s10654-014-9930-3

[65] Ponten JEH, Leclercq WKG, Lettinga T, Heemskerk J, Konsten JLM, Bouvy ND, et al. Mesh OR Patch for Hernia on Epigastric and Umbilical Sites (MORPHEUS-Trial): The Complete Two-year Follow-up. Ann Surg. 2019;270(1):33–7. doi: 10.1097/SLA.0000000000003086 30339623

[66] Winsnes A, Haapamäki MM, Gunnarsson U, Strigård K. Surgical outcome of mesh and suture repair in primary umbilical hernia: postoperative complications and recurrence. Hernia. 2016;20(4):509–16. doi: 10.1007/s10029-016-1466-x 26879081

[67] Helgstrand F, Rosenberg J, Kehlet H, Strandfelt P, Bisgaard T. Reoperation versus clinical recurrence rate after ventral hernia repair. Ann Surg. 2012;256(6):955–8. doi: 10.1097/SLA.0b013e318254f5b9 22580941

[68] Berger RL, Li LT, Hicks SC, Davila JA, Kao LS, Liang MK. Development and validation of a risk-stratification score for surgical site occurrence and surgical site infection after open ventral hernia repair. J Am Coll Surg. 2013;217(6):974–82. doi: 10.1016/j.jamcollsurg.2013.08.003 24051068

[69] Morrison BG, Gledhill K, Plymale MA, Davenport DL, Roth JS. Comparative long-term effectiveness between ventral hernia repairs with biosynthetic and synthetic mesh. Surg Endosc. 2023;37:6044–50. 37118030 10.1007/s00464-023-10082-1

[70] Henriksen NA, Bisgaard T, Helgstrand F, Danish Hernia Database. Smoking and obesity are associated with increased readmission after elective repair of small primary ventral hernias: A nationwide database study. Surgery. 2020;168(3):527–31. doi: 10.1016/j.surg.2020.04.012 32460998

[71] Liljendahl M, Grønbæk AS, Jensen AB, Tønnesen H. The STOPbase year report: activities in 2022 with follow-up in 2023. Appendix. 2024 [cited February 25, 2025]. https://www.stopbasen.dk/%C3%A5rsrapport-2023

[72] Ohälsosamma matvanor [Internet]. Allmänläkarkonsult Region Skåne; 2024 [cited February 25, 2025]. https://vardgivare.skane.se/vardriktlinjer/levnadsvanor/ako/ohalsosamma-matvanor/#252800

[73] van Gils Y, Franck E, Dierckx E, van Alphen S, Saunders J, Dom G. Validation of the audit and audit-c for hazardous drinking in community-dwelling older adults. Int J Environ Res Public Health. 2022;18:9266. 10.3390/ijerph18179266 PMC8431181 34501856

[74] Takahashi T, Lapham G, Chavez LJ, Lee AK, Williams EC, Richards JE, et al. Comparison of DSM-IV and DSM-5 criteria for alcohol use disorders in VA primary care patients with frequent heavy drinking enrolled in a trial. Addict Sci Clin Pract. 2017;12(1):17. doi: 10.1186/s13722-017-0082-0 28716049 PMC5514480

[75] Sullivan JT, Sykora K, Schneiderman J, Naranjo CA, Sellers EM. Assessment of alcohol withdrawal: the revised clinical institute withdrawal assessment for alcohol scale (CIWA-Ar). Br J Addict. 1989;84(11):1353–7. doi: 10.1111/j.1360-0443.1989.tb00737.x 2597811

[76] Charlson ME, Carrozzino D, Guidi J, Patierno C. Charlson Comorbidity Index: A Critical Review of Clinimetric Properties. Psychother Psychosom. 2022;91(1):8–35. doi: 10.1159/000521288 34991091

[77] Clavien PA, Barkun J, de Oliveira ML, Vauthey JN, Dindo D, Schulick RD, et al. The Clavien-Dindo classification of surgical complications: five-year experience. Ann Surg. 2009;250(2):187–96. doi: 10.1097/SLA.0b013e3181b13ca2 19638912

[78] ASA House of Delegates. ASA Physical Status Classification System. n.d. [cited February 25, 2025]https://www.asahq.org/standards-and-guidelines/asa-physical-status-classification-system

[79] Malterud K. Systematic text condensation: a strategy for qualitative analysis. Scand J Public Health. 2012;40(8):795–805. doi: 10.1177/1403494812465030 23221918

[80] WHO. WHO report on the global tobacco epidemic 2019: offer help to quit tobacco use. n.d. [cited February 25, 2025] https://www.who.int/publications/i/item/9789241516204

[81] World Health Organization. Integrated brief interventions for noncommunicable disease risk factors in primary care: the manual: BRIEF project. 2022 [cited February 25, 2025]. https://www.who.int/europe/publications/i/item/9789289058551

[82] Hovhannisyan K, Rasmussen M, Adami J, Wikström M, Tønnesen H. Evaluation of Very Integrated Program: Health Promotion for Patients With Alcohol and Drug Addiction-A Randomized Trial. Alcohol Clin Exp Res. 2020;44(7):1456–67. doi: 10.1111/acer.14364 32424821

[83] Lauridsen SV, Thomsen T, Kaldan G, Lydom LN, Tønnesen H. Smoking and alcohol cessation intervention in relation to radical cystectomy: a qualitative study of cancer patients’ experiences. BMC Cancer. 2017;17(1):793. doi: 10.1186/s12885-017-3792-5 29178899 PMC5702236

[84] Tønnesen H, Lydom LN, Joensen UN, Egerod I, Pappot H, Lauridsen SV. STRONG for Surgery & Strong for Life - against all odds: intensive prehabilitation including smoking, nutrition, alcohol and physical activity for risk reduction in cancer surgery - a protocol for an RCT with nested interview study (STRONG-Cancer). Trials. 2022;23(1):333. doi: 10.1186/s13063-022-06272-2 35449008 PMC9027477

